# Sex Differences in the Variability of Physical Activity Measurements Across Multiple Timescales Recorded by a Wearable Device: Observational Retrospective Cohort Study

**DOI:** 10.2196/66231

**Published:** 2025-04-28

**Authors:** Kristin J Varner, Lauryn Keeler Bruce, Severine Soltani, Wendy Hartogensis, Stephan Dilchert, Frederick M Hecht, Anoushka Chowdhary, Leena Pandya, Subhasis Dasgupta, Ilkay Altintas, Amarnath Gupta, Ashley E Mason, Benjamin L Smarr

**Affiliations:** 1 Shu Chien-Gene Lay Department of Bioengineering University of California San Diego La Jolla, CA United States; 2 UC San Diego Health Department of Biomedical Informatics University of California San Diego La Jolla, CA United States; 3 Department of Bioinformatics and Systems Biology University of California San Diego La Jolla, CA United States; 4 Osher Center for Integrative Health University of California San Francisco San Francisco, CA United States; 5 Department of Management Zicklin School of Business Baruch College, The City University of New York New York, NY United States; 6 San Diego Supercomputer Center University of California San Diego La Jolla, CA United States; 7 Halıcıoğlu Data Science Institute University of California San Diego La Jolla, CA United States

**Keywords:** wearables, activity, sex as a biological variable, time series variance, timescales of change, metabolic equivalents, metabolic equivalent of task, sex differences

## Abstract

**Background:**

A substantially lower proportion of female individuals participate in sufficient daily activity compared to male individuals despite the known health benefits of exercise. Investment in female sports and exercise medicine research may help close this gap; however, female individuals are underrepresented in this research. Hesitancy to include female participants is partly due to assumptions that biological rhythms driven by menstrual cycles and occurring on the timescale of approximately 28 days increase intraindividual biological variability and weaken statistical power. An analysis of continuous skin temperature data measured using a commercial wearable device found that temperature cycles indicative of menstrual cycles did not substantially increase variability in female individuals’ skin temperature. In this study, we explore physical activity (PA) data as a variable more related to behavior, whereas temperature is more reflective of physiological changes.

**Objective:**

We aimed to determine whether intraindividual variability of PA is affected by biological sex, and if so, whether having menstrual cycles (as indicated by temperature rhythms) contributes to increased female intraindividual PA variability. We then sought to compare the effect of sex and menstrual cycles on PA variability to the effect of PA rhythms on the timescales of days and weeks and to the effect of nonrhythmic temporal structure in PA on the timescale of decades of life (age).

**Methods:**

We used minute-level metabolic equivalent of task data collected using a wearable device across a 206-day study period for each of 596 individuals as an index of PA to assess the magnitudes of variability in PA accounted for by biological sex and temporal structure on different timescales. Intraindividual variability in PA was represented by the consecutive disparity index.

**Results:**

Female individuals (regardless of whether they had menstrual cycles) demonstrated lower intraindividual variability in PA than male individuals (Kruskal-Wallis H=29.51; *P*<.001). Furthermore, individuals with menstrual cycles did not have greater intraindividual variability than those without menstrual cycles (Kruskal-Wallis H=0.54; *P*=.46). PA rhythms differed at the weekly timescale: individuals with increased or decreased PA on weekends had larger intraindividual variability (Kruskal-Wallis H=10.13; *P*=.001). In addition, intraindividual variability differed by decade of life, with older age groups tending to have less variability in PA (Kruskal-Wallis H=40.55; *P*<.001; Bonferroni-corrected significance threshold for 15 comparisons: *P*=.003). A generalized additive model predicting the consecutive disparity index of 24-hour metabolic equivalent of task sums (intraindividual variability of PA) showed that sex, age, and weekly rhythm accounted for only 11% of the population variability in intraindividual PA variability.

**Conclusions:**

The exclusion of people from PA research based on their biological sex, age, the presence of menstrual cycles, or the presence of weekly rhythms in PA is not supported by our analysis.

## Introduction

### Background

Regular physical activity (PA) compared to inactivity is associated with a lower risk of all-cause mortality in both male and female individuals [1]. Nevertheless, a meta-analysis reported that PA decreased in several countries between 1995 and 2017 [2]. While this decrease has occurred equally in both male and female individuals, female individuals are less likely to participate in sufficient exercise [3-5]. An evaluation of insufficient activity (defined as participating in <150 min of moderate-intensity or <75 min of vigorous-intensity PA per wk) among 1.9 million participants found that 27.5% did not participate in sufficient activity; moreover, women had significantly higher rates of inactivity than men (31.7% vs 23.4%) [3]. As female individuals have been shown to derive greater risk reduction than male individuals for an equivalent increase in exercise [1], it is important to identify the causes of the sex or gender gap in PA. While the reasons for this gap are not well understood [5], it has been attributed to many factors, including children’s exposure to rigid gender norms; women’s concerns about stereotypes; a lack of leisure time; and, importantly, a lack of investment in women’s and girls’ sports [4]. These knowledge gaps pervade sports and exercise science research. An analysis of 3 major sports and exercise medicine journals over 3 years (2011-2013) found that just 39% of participants in 1382 original research articles were female [6]. A subsequent analysis of 5621 studies from 6 sports and exercise journals (including the 3 journals in the previous study) examined research published over the next 7 years (2014-2020) and reported a lower proportion of total female participants (34%) and a significantly higher number of studies including only male (1631/5261, 31%) versus only female (328/5261, 6.23%) participants [7]. The exclusion of female participants from sports and exercise medicine studies is partly attributed to the assumption that ovarian hormones (or menstrual cycles) increase intraindividual PA variability in female individuals, thereby increasing the difficulty in interpreting the results (due to increased intraindividual variability contributing to greater interindividual variability) or complicating methodology to account for changes in ovarian hormones [8-11]. This assumption further suggests that the results obtained from male participants are generalizable to female participants: if male and female baseline physiology is the same but female participants exhibit greater intraindividual variability, their inclusion would merely increase population-level (interindividual) variability, reduce statistical power, and offer no benefit to the study. However, the hypothesis that the results obtained from male participants are generalizable to female participants (or that they have the same baseline physiology) has repeatedly been shown to be false [1,12-14]. This in itself should motivate the inclusion of female participants, but as female participation in sports and exercise research is still low compared to male participation [6,7], it is important to assess the extent to which menstrual cycles and other biological and social rhythms interfere with researchers’ ability to analyze PA. Building on previous work exploring physiological variability from distal skin temperature measured by a commercial wearable device [15], in this study, we explore the intraindividual variability in PA between the sexes using longitudinal PA measurements from 596 individuals (male: n=298, 50%; female: n=298, 50%) who were using Oura Rings in 2020.

Numerous animal studies have rejected the hypothesis that female animals are more variable in both physiology and behavior [16-19], but far fewer studies have examined whether this pattern holds in humans [15,20]. This is in part due to historical difficulty in generating longitudinal datasets that are sufficiently large to be representative of both sexes broadly. The emergence of digital tools such as wearable devices in daily life has led to a rapid change in the amount of longitudinal data that can be easily collected from individual study participants. Data from wearables provide unique opportunities to explore physiological and behavioral variability between sexes both across populations and within individual time series data [21].

In our previous work, we used continuous longitudinal distal skin temperature data generated by Oura Ring users in situ to test the hypothesis that female individuals are statistically more physiologically variable than male individuals [15]. Temperature was chosen because prior work indicates that skin temperature can be used to identify physiological changes, such as a 28-day oscillating skin temperature pattern generated by menstrual cycles [22,23]. Using a dataset of minute-level skin temperature data from 600 individuals (male: n=300, 50%; female: n=300, 50%) over 6 months, we developed a tool capable of determining cyclic status, where female individuals’ data that showed an approximately 28-day pattern in nightly maximum temperature were labeled as cyclic, and those without were labeled as acyclic. We also found that cyclic individuals and acyclic individuals of either sex, showed substantially different patterns of change over time such that cyclic status was a more informative label than sex when predicting the structure of variability in an individual’s skin temperature over time. Our analyses led us to reject the hypothesis that female individuals, whether cyclic or acyclic, should be excluded due to concerns over statistical power, although our findings also supported the use of sex as a biological variable (SABV) in analyses (ie, body temperature changes linked to menstrual cycles [24] were present in a subset of individuals who self-reported as biologically female). While the variability was not substantially greater at multiple timescales in any of these groups, the means and temporal structure of temperature predictably differed by biological sex and cyclic status. In this study, we seek to recapitulate these analyses on the same population but focus on PA because it is less closely tied to hormonal changes physiologically and instead more reflective of behavioral changes.

Previous studies have demonstrated that multiple timescales of change can interact to give rise to nonrandom structure in intraindividual variability of human time series data [15,20,25]. This temporal structure arises specifically from interactions between physiological rhythms such as menstrual and circadian rhythms, societal phenomena such as the 7-day work week, and nonrhythmic temporal scales such as aging. To the extent that variability is nonrandom, it is by definition at least partially predictable. If not accounted for in experimental design, then nonrandom (unaccounted) variability will be combined with random (unaccountable) variability to the effect that statistical tests—by treating all sources of variability as equivalent—will yield reduced power for detecting real effects. By contrast, when nonrandom variability is accounted for, residual variability is by definition lower, and statistical power is improved for the same analysis. Although the sources and structures of male variability are not well characterized [13], the impact of these other timescales of change on variability is not often considered; without a direct comparison, we cannot know how these other timescales of change influence PA analyses compared to the effects of menstrual cycles.

### Objectives

In this study, we used the same cohort of participants as in our previous analysis of temperature [15] to assess the effect of sex, cyclic status, and temporal structures in PA on other timescales of change on intraindividual PA variability. Specifically, we sought to determine whether the presence of approximately 28-day cyclic temperature patterns we previously identified correlates with increased intraindividual variability in PA measurements and to quantify the extent that these approximately 28-day cycles affect statistical analysis of PA. In addition, we sought to ascertain whether temporal structure occurring on other timescales besides menstrual cycles (eg, weeks and decades) contribute to intraindividual PA variability. The Oura Ring reports activity in the form of metabolic equivalents of tasks (METs) [26], where METs express the intensity of an activity as multiples of the MET recorded at rest [27]. Using these measurements, we quantified individual daily PA and intraindividual variability in PA and found that biological sex, cyclic status, and weekly and decadal temporal structures in PA do not explain most of the intraindividual variability in PA.

## Methods

### Data Source

Data originated from the TemPredict Study [26]. Physiological data were collected using the Oura Ring (Oura Health Oy, Oulu, Finland), and self-reported demographic information such as sex and age were collected via survey.

### Participants

Participants were identified by using the filtering methods described in the study by Bruce et al [15]. Briefly, 62,653 participants were determined to have suitable physiological and demographic data. Responses to the survey question “What is your biological sex? Male, Female, Other (please describe)” were used to determine participants’ sex.

Filtering for participants with data files for all data types and for whom temperature data were available for all months between January and November 2020 led to the exclusion of 54,738 (87.37%) of the 62,653 participants, leaving 7915 (12.63%) participants. Next, participants who had <70% average daily completeness in temperature data were excluded. We chose to filter out participants with <70% average daily completeness in temperature data to increase the likelihood that both sleep and wake states were captured in the data (sleep usually covers approximately 33% of a day). A cohort of 600 individuals (female: n=300, 50%; male: n=300, 50%) was chosen from the final list such that 50 (16.7%) of the 300 individuals of each sex were present in six 10-year age bins spanning 20 to 79 years.

Additional filtering of the participants was performed for this analysis. The lower limit for real MET recordings is 0.9, which corresponds to a person being asleep [28]. All MET values of <0.9 were dropped (due to non–wear time artifacts), and participants were evaluated for missingness over 206 days between April and October 2020. In total, 4 participants, 2 (50%) of each sex, with a percentage missingness of MET data of >29% were removed (Figure S1 in Multimedia Appendix 1). The final data consisted of 206 consecutive days for 596 individuals (female: n=298, 50%; male: n=298, 50%). Six age bins were represented equally with 49 (16.4%) to 50 (16.8%) of the 298 individuals of each sex in each age bin: 20 to 29, 30 to 39, 40 to 49, 50 to 59, 60 to 69, and 70 to 79 years.

### Data Preprocessing

High-resolution (per 1 min and per 5 min) and nightly aggregated data were generated by the Oura Ring. Data were stored in large parquet files on a server hosted at the San Diego supercomputer and accessed through the Nautilus portal [29]. We expected METs to vary by sleep state (whether an individual is awake or asleep); therefore, we labeled minute-level data with asleep and awake labels. Nightly data, also referred to as sleep summary data, were stored as a single parquet file for each participant. These data contained sleep-related information such as sleep time start and sleep time end. The longest sleep duration for each day was used to label measurements as *asleep*. All other times were labeled as *awake*.

High-resolution distal body temperature and MET data were recorded at 1-minute intervals for 24 hours per day. These data were date-time indexed and normalized to participants’ local time. Duplicate time points were removed, and the remaining time points were annotated as awake or asleep.

METs were calculated by Oura Ring before data were transferred to us for analysis. Triaxial accelerometers were used to estimate METs at 1-minute resolution during both sleep and wake periods [26]. The exact MET calculation used by the Oura Ring is proprietary and not disclosed to us; however, Oura Ring (Gen 2) activity measurements displayed high correlation when validated against multiple accelerometers [30].

### Data Filling

Missing sleep state data and MET data were filled for all 596 participants. Sleep state data described the sleep state (awake or asleep) at every minute for every participant. MET data contained the MET value at every minute for every participant.

To limit the artifacts resulting from data filling, we assessed the accuracy of 4 filling methods on several intervals of missingness. An interval of missingness describes the number of consecutive minutes for which there are missing values (ie, an interval of 1440 describes a full missing day). The intervals tested were 5, 10, 20, 40, 80, 160, 320, 640, 1280, and 1440 minutes. The filling methods tested were (1) a phase-dependent filler, (2) linear interpolation, (3) global personal median filling, and (4) zero filling (or “not a number” filling). A detailed description of each method is provided in the following list:

1. The phase-dependent filler constructs a “median week” from the median value of each minute on each day of the week across half of the dataset (103 d) for each participant (2 median wk per participant). If no median value exists for a minute in the constructed median week, a value was forward-filled from the median value of the preceding minute. The minutes without data in the 103-day period from which the week of median values was constructed were filled based on the minute and day of the week in which they occurred.
2. Linear interpolation was achieved with the interpolate method from the Python package *pandas* (*pandas.DataFrame.interpolate*, version 2.2.1 [31]). A 2-way limit direction was used such that missing data from the first minute in the data could be filled.
3. The global personal median filling finds the median value for each person across the entire dataset and fills the missing values with this median value.
4. The zero-filling method fills all missing values with 0. This method was included because the sum of MET values was used to summarize daily activity. Zero fill equates to the effect of not filling these values because “not a number” is treated as 0 during daily summation.

To test the accuracy of the filling methods for each interval length, a test data frame was constructed. For each participant, simulated missing data were constructed by inserting intervals of missingness starting at randomly chosen minutes. Each participant had 3995 extra missing data points composed of intervals of 5, 10, 20, 40, 80, 160, 320, 640, 1280, and 1440 minutes of missingness. The intervals were allowed to overlap and occur on the same day. The simulated intervals of missingness were then filled using each of the 4 filling methods. After filling, the predicted values in the sleep state data frame were rounded to 0 or 1 to reflect a prediction of being asleep or awake, respectively.

The performance of each method for each person on each interval size was evaluated by the sum of the absolute differences between the predicted and actual values of the test indexes. As some participants did not have enough data, some simulated missing data had indeterminate error (the “actual” value was missing): 0.25% of the simulated missing data in the MET filling test had indeterminate error, and 0.49% of the simulated missing data in the sleep state filling test had indeterminate error. The best method for each interval size was determined by the smallest sum of absolute differences across all individuals. In the MET dataset, the best method for filling intervals of missingness of ≤40 minutes was linear interpolation, and for intervals of >40 minutes, the best method was phase-dependent filler (error data shown in Figure S2 in Multimedia Appendix 1). In the sleep state dataset, the best method for intervals of missingness of ≤320 minutes was linear interpolation, and for intervals of missingness of >320 minutes, the best method was phase-dependent filler (error data shown in Figure S3 in Multimedia Appendix 1). The best filling method for each interval of missingness was applied to each dataset before any analyses were performed.

The sum of absolute differences across all test intervals (filling error) was not significantly different between male participants, cyclic female participants, and acyclic female participants in the sleep state and MET data tests (Kruskal-Wallis test, MET data: H=1.97; *P*=.37; Figure S4 in Multimedia Appendix 1; sleep state: H=0.26; *P*=.88; Figure S5 in Multimedia Appendix 1).

Filled data were used for every analysis described herein, except where explicitly stated otherwise (refer to the Analysis by Weekend Rhythm in PA subsection).

### Statistical Methods

#### Kruskal-Wallis H Tests, Bonferroni Correction, and Post Hoc Dunn Tests

**Figure 1:**
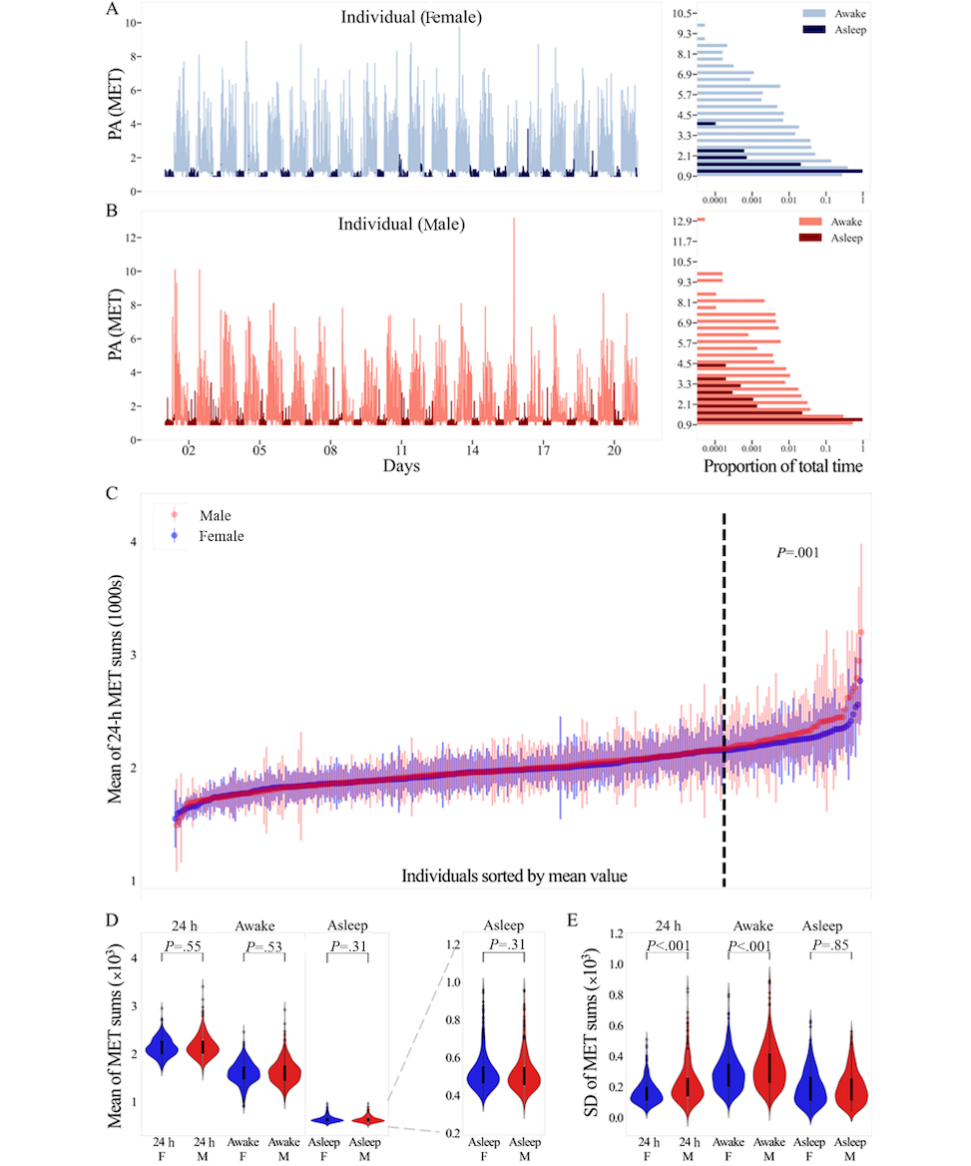
Longitudinal plot of a representative 3-week interval of minute-level metabolic equivalent of task (MET) data (left) from (A) 1 female individual (F, blue) and (B) 1 male individual (M, red), with the histogram of the MET values for each separated by awake (light) and asleep (dark) values (right). MET values were examined at minute-level resolution. Histograms show the percentage time (percentage time is shown on a log scale and referenced in the figure as “Proportion of total time”) spent in 37 bins of MET values while awake or asleep. MET values range from 0.9 to 16.1, and each bin is 0.4 METs in size. (C) Plot of all individuals’ (n=596) mean (dot) and SD (vertical line) of 24-hour daily MET sums, sorted by mean. The dashed line separates the 60 individuals in each sex with the largest means from the rest of the population. The top 60 were subsequently compared across sex (Kruskal-Wallis test). (D) Violin plots of male and female individuals’ means and (E) SDs for 24-hour MET sums, awake time state MET sums, and asleep time state MET sums (Kruskal-Wallis test; Bonferroni-corrected significance threshold for 3 comparisons: *P*=.02). PA: physical activity.

**Figure 2:**
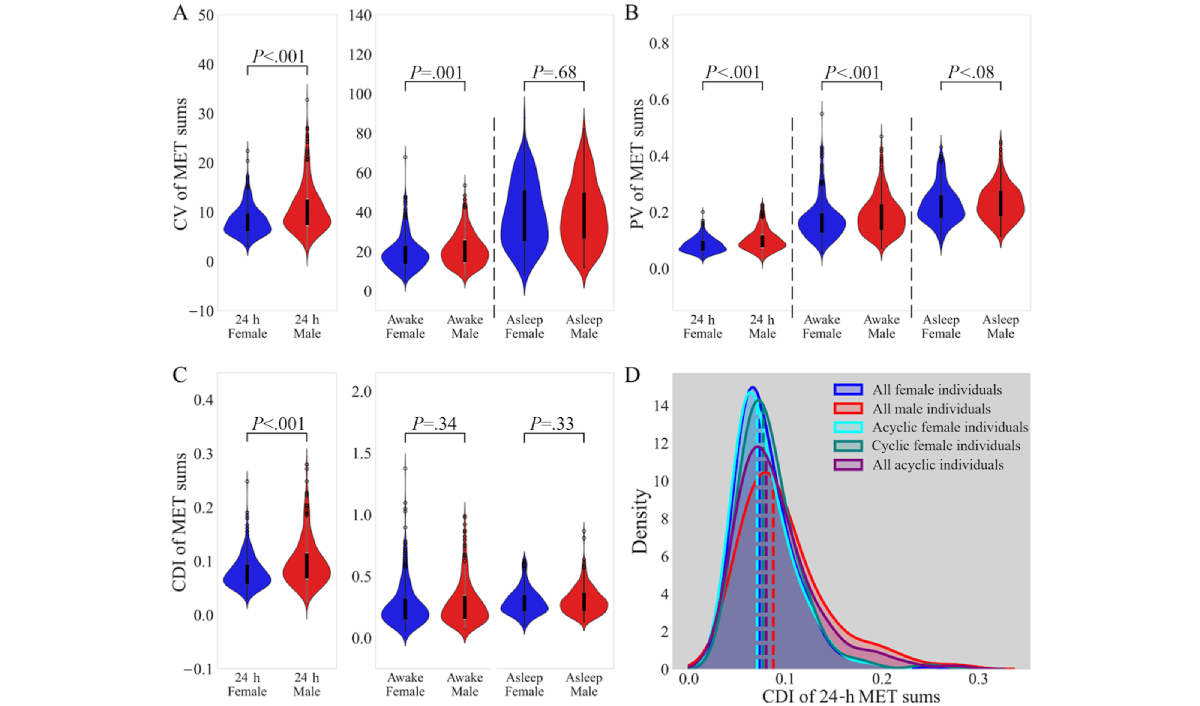
Violin plots showing the distributions of (A) coefficient of variation (CV), (B) proportional variability index (PV), and (C) consecutive disparity index (CDI) for 24-hour metabolic equivalent of task (MET) sums, awake time state MET sums, and asleep time state MET sums for female (blue) and male (red) individuals (Kruskal-Wallis test, Bonferroni-corrected significance threshold for 3 comparisons: *P*=.02). (D) Kernel density estimate plots of sex-cyclic, sex, and cyclic groups. Group median CDI values are indicated by dashed vertical lines.

**Figure 3:**
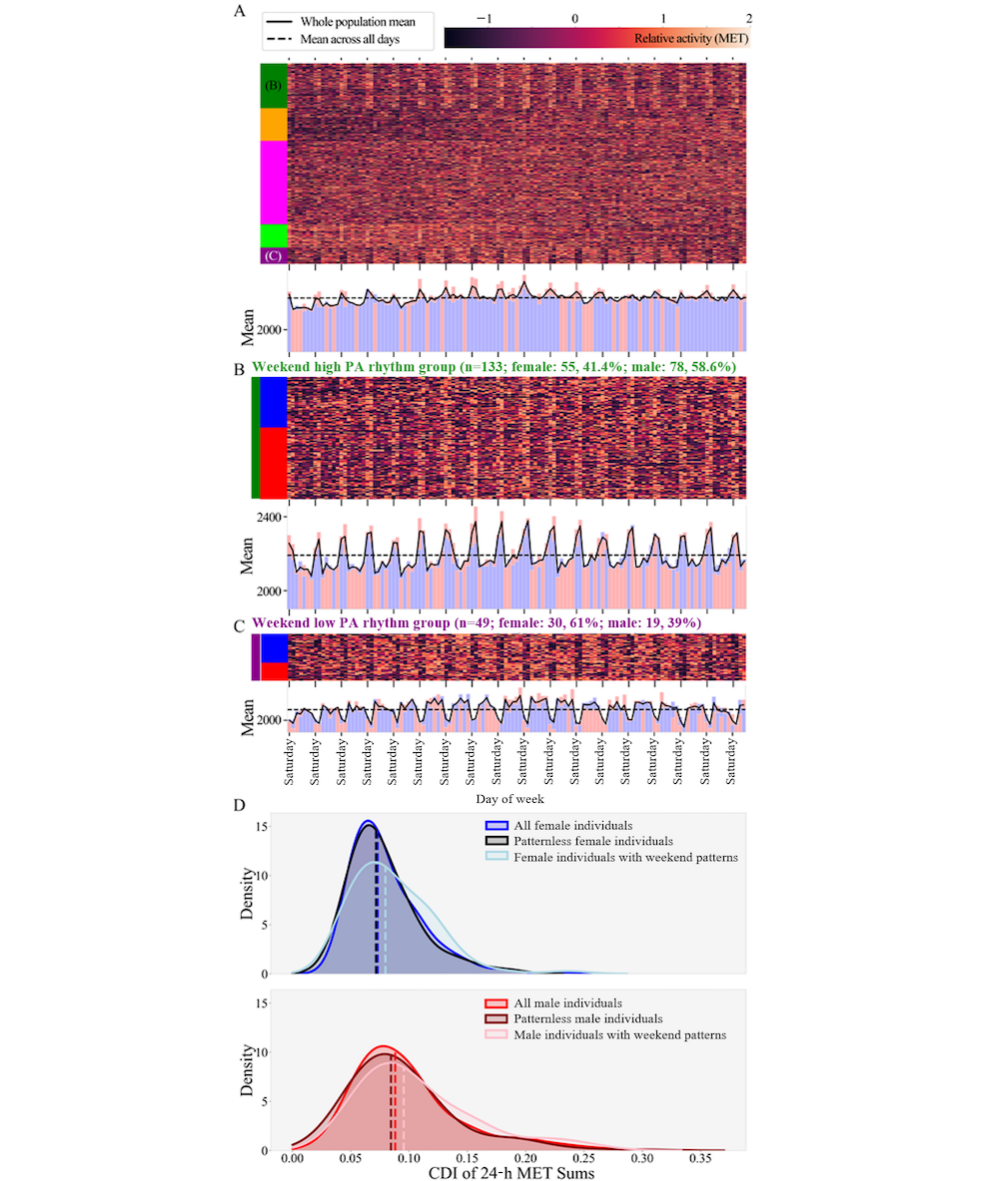
(A) Heat map of relative activity, expressed using the daily 24-hour metabolic equivalent of task (MET), for every individual across 4 consecutive months. Relative activity was defined as arctan (2 × intraindividual z score of daily 24-h MET sums). Relative activity values of >2 and <−1.5 are shown in the lightest and darkest colors, respectively. Individuals are sorted by agglomerative cluster number, and clusters are demarcated by the colors in the bar to the left of the heat map. The line and layered bar plot below each heat map shows the daily mean 24-hour MET sum across all individuals in the connected heat map (solid black line), the mean 24-hour MET sum across all days in the 4-month period (dashed black line), and the daily 24-hour MET sum mean of the male (red) and female (blue) individuals where the sex with the lower mean for each day was layered on top. (B) Magnified heat map of the dark green cluster: weekend high physical activity (PA) rhythm group. (C) Magnified heat map of the dark purple cluster: weekend low PA rhythm group. Heat map rows, representing 1 individual each, are all of equal size so that the height of the heat map is representative of the number of people in the cluster. Individuals are labeled and sorted by sex (blue box on the left of the heat map for female individuals and red box for male individuals). (D) Kernel density estimate plot of consecutive disparity index (CDI) calculated from 4 consecutive months for the female and male whole population, the weekend cluster population, and other clusters. Vertical dashed lines represent the population median CDI.

**Figure 4:**
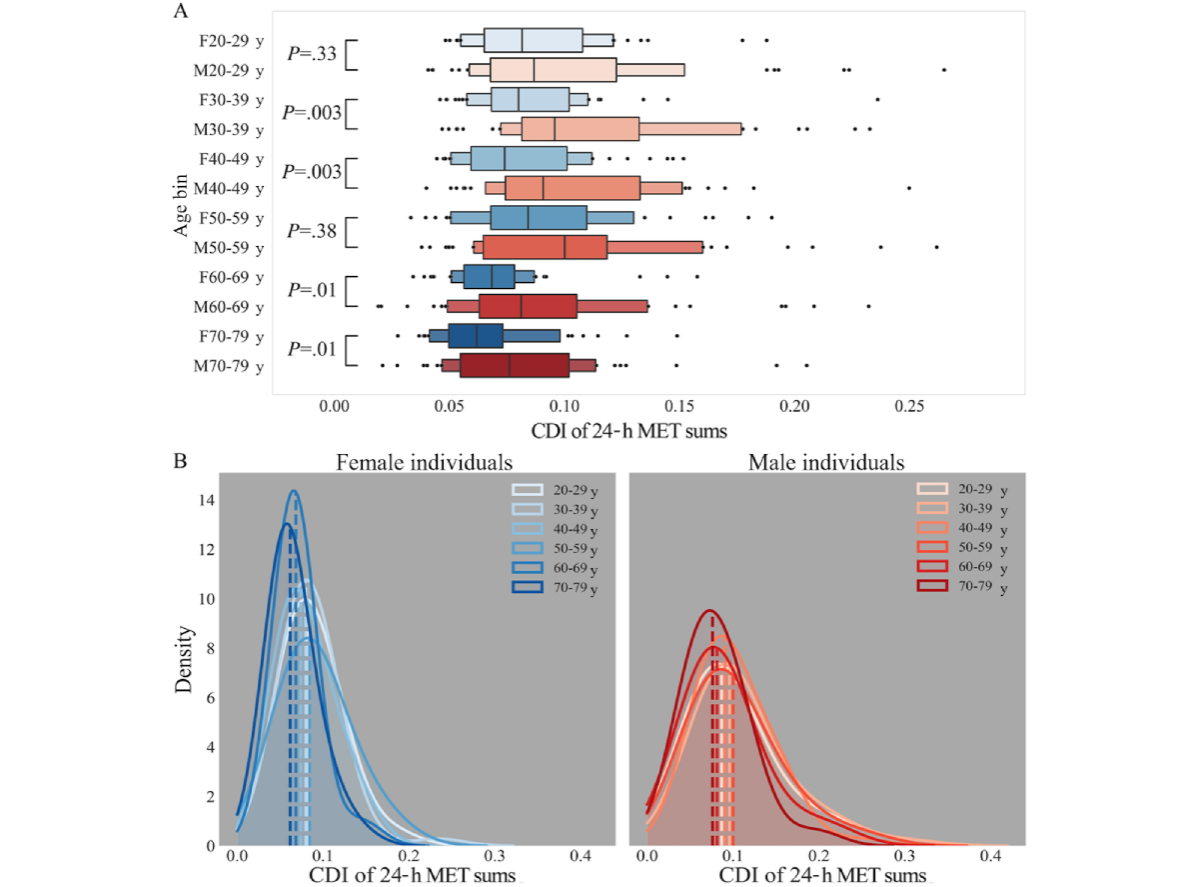
(A) Boxen plot of consecutive disparity indices (CDIs) for each sex (F: Female individuals; M: Male individuals) and age bin (year range) (Kruskal-Wallis test, Bonferroni-corrected significance threshold for 6 comparisons: *P*=.008). (B) Kernel density estimate plots of CDI in each age bin for female and male individuals. Dashed lines indicate the median CDI of the sex-age population. MET: metabolic equivalent of task.

Population differences were determined using a Kruskal-Wallis H test between population distributions of the relevant metric (mean, SD, etc). Python was used to carry out all Kruskal-Wallis tests (SciPy library: *scipy.stats.kruskal*, version 1.11.2 [32]). In the case that ≥3 populations were compared, a Bonferroni correction was manually applied to all analyses that compared >2 groups such that the threshold for significance (*P*=.05) was divided by the number of comparisons made. If the significance threshold was met, and groups were compared with a single Kruskal-Wallis test, a post hoc Dunn test was performed using Python (*scikit_posthocs.posthoc_dunn*, version 0.9.0 [33]) to identify the pair-wise population comparisons that met the significance threshold. Although the shape of distributions for male participants tended to be wider than that of distributions for female participants, median values were used to determine the population with the larger metric. The results from these tests and the distributions compared with these tests are shown in most of the figures and tables (Figures 1C-E, 2A-D, 3D, and 4A and 4B; Figures S2-S5 in Multimedia Appendix 1). Population SDs of the subpopulations described were calculated for their relevance to power analysis (Tables S1 and S2 in Multimedia Appendix 1).

#### Modified Cohen *d* Effect Size

As the distributions in these analyses were nonnormal, a modified Cohen *d* effect size (Cohen *d_m_*) was used to describe the magnitude of the difference between 2 significantly different populations (shown as P1 and P2 here) [34]:

*d_m_* = (|median(P1) – median(P2)|) / (mean(IQR(P1), IQR(P2))) **(1)**

where IQR(P1) and IQR(P2) represent the IQRs of the populations (IQR=the difference between the 75th and 25th percentile values). This modification to the Cohen *d* effect size compares medians instead of means and IQRs instead of SDs to accommodate calculations appropriate for skewed distributions.

The Cohen *d_m_* effect size approximates the proportion of population variability accounted for by a characteristic (sex, age, etc); for example, if Cohen *d_m_*=1, the difference in the medians is equal to the mean of the 2 population IQRs, which means that there is little overlap of values, and the characteristic accounts for a substantial proportion of the variability between these populations. Cohen *d_m_* was calculated between subpopulations that were significantly different by either a Kruskal-Wallis or a post hoc Dunn test (Figures 1C, 2C and 2D, 3D, and 4B).

#### Effect of Subpopulations

To determine whether a subpopulation contributes a significant amount of variability to a whole population, we first identified 2 groups of participants: the whole population and the whole population excluding the subpopulation of interest. The second group is itself a subset of the whole population, which makes statistical comparisons problematic: the whole population contains every value in the subset. To avoid making comparisons between identical values, we calculated the IQRs of the 24-hour MET sums for each day for each group. This generated 2 lists of 206 IQRs representing each group’s variability across the 206 days in this study. The 2 lists were compared with a Kruskal-Wallis test to evaluate whether a whole population changed when a subpopulation was excluded. If the whole population had significantly larger IQRs than the whole population with the subpopulation of interest excluded, then the subpopulation was considered to have imparted a significant amount of variability on the whole population. This test was performed on the distributions shown in Figures 2D and 3D. If a subpopulation did impart a significant amount of variability on the whole population, we used the rule formulated by Lehr [35] to calculate the difference in sample size required to detect the same effect (with 80% power and a significance level of .05) when the group was included or excluded:


n = 16(s^2^) / (µ_1_ − µ_2_)^2^
**(2)**

where n is the sample size required, s^2^ is the variance of the population tested, and (µ_1_ − µ_2_) is the difference in means between each population. We used the median IQR across all 206 days as a proxy for s and tested multiple values for (µ_1_ − µ_2_): 40 (approximately the difference in 24-hour MET sums resulting from a 20-min walk), 100 (approximately the difference in 24-hour MET sums resulting from 20 min of moderate-intensity activity), and 180 (approximately the difference in 24-hour MET sums resulting from 20 min of high-intensity activity). We chose these values to represent a difference that may be significant to health.

#### Kernel Density Estimate Plots

Kernel density estimate plots were used to ensure that distributions were visually comparable despite differences in group size and to enable comparisons of idealized distributions. Plotting was performed in Python using the *seaborn* library (*seaborn.kdeplot*, version 0.12.2 [36]) with the default kernel (Gaussian) and bandwidth smoothing method (the Scott rule). The bandwidth scaling parameter (bw_adjust) was adjusted per distribution to create visually smoother plots, and estimation ranges were limited to real values. Kernel density estimate plots are displayed in Figures 2D, 3D, and 4B.

### Cohort and MET Data Foundational Analysis

To visually inspect the effect of time of day on activity, a random subset of 20 consecutive days of data from 2 randomly selected individuals of each sex was chosen to represent a MET value time series and distribution (Figures 1A and B). Finding that MET values were highly dependent on awake or asleep state as expected, we summed MET values for each day (206 d in total) over 24 hours, awake time states, and asleep time states to summarize the total daily PA for each person in each state. These states were considered separately in subsequent analyses because the source of the variability of daily MET sums is different in each state. We considered 5 drivers of variability: awake movement, intentional exercise, sleep movement, time spent asleep, and time spent awake. The first 3 drivers of variability are associated with a state (awake or asleep) and a MET range. Sleep movement occurs while asleep and at a MET value of >0.9 (sleep results in a MET value of 0.9 [28]), awake movement occurs while awake and at a MET value of between 1.0 and 1.5 (resting while awake results in a MET value of 1.0, and intentional exercise results in a MET value of >1.5 [28]), and intentional exercise occurs while awake and at a MET value of >1.5. Time spent awake and time spent asleep refer to the number of minutes per day that a person spends awake and asleep. In contrast to 24-hour MET sums, where the number of values being summed is always 1440 (24 h × 60 min), awake and asleep daily MET sums vary by the number of values being summed per day due to varying amounts of time spent in these states each day. The possible sources of variability in 24-hour sums are sleep movement, awake movement, and intentional exercise. The possible sources of variability in awake daily sums are time spent awake, intentional exercise, and awake movement. The possible sources of variability in asleep daily sums are sleep duration and movement while asleep.

A PA summary of all participants across all 206 days was constructed from the means and SDs of the 206 daily 24-hour MET sums. Individuals in each sex population were sorted by the mean of 24-hour MET sums and represented as a point and line representing +1 or −1 intraindividual SD such that individuals at the same rank in each population could be compared. Noticing a divergence between the populations in the individuals with the largest means, we performed a Kruskal-Wallis test between the top 60 male participants and the top 60 female participants (Figure 1C).

Whole-population distributions of mean and SD values for male and female participants across all 206 days for 24-hour, awake, and asleep MET sums were compared using a Kruskal-Wallis test with a Bonferroni correction for 3 comparisons (3 MET sum metrics each for mean and SD; Figures 1D and E).

### Variability Metrics of MET Sums

#### Overview

In addition to SD, we used 3 other metrics to analyze intraindividual variability: coefficient of variation (CV), proportional variability index (PV), and consecutive disparity index (CDI). In prior work, we used CV and PV as controls to validate the statistical findings from the CDI analyses [15]. We included CV and PV in this study for the same validation and focused on CDI because it is the most appropriate metric of intraindividual variability for these data because it accounts for chronological order and is not dependent on the mean for its calculation. Further analyses used only CDI as a variability metric. Whole-population distributions of CV, PV, and CDI values for male and female participants across all 206 days for 24-hour, awake, and asleep MET sums were compared using a Kruskal-Wallis test with a Bonferroni correction for 3 comparisons (3 MET sum metrics each for CV, PV, and CDI).

#### CV Metric

CV is a common metric for describing temporal variability [37]. In this study, it describes a participant’s SD (σ) across all 206 days compared to their mean across all 206 days:


CV = σ / mean **(3)**

CV is limited by its sensitivity to rare events and its dependence on the mean [37] (Figure 2A).

#### PV Metric

The PV was developed to solve some of the limitations of CV. The PV quantifies variability by calculating the average percentage difference between all combinations of measurements [37-40]:


PV = 2(Σ(1-(min(z_i_, z_j_) / max(z_i_, z_j_))) / n(n-1) **(4)**

where n is the total number of values, z is a list of values on which pair-wise comparisons are calculated, and i and j are indices of any 2 different values. The PV improves upon CV because it is not mean dependent, and it is less sensitive to rare events [37] (Figure 2B).

#### CDI Metric

**Figure 5:**
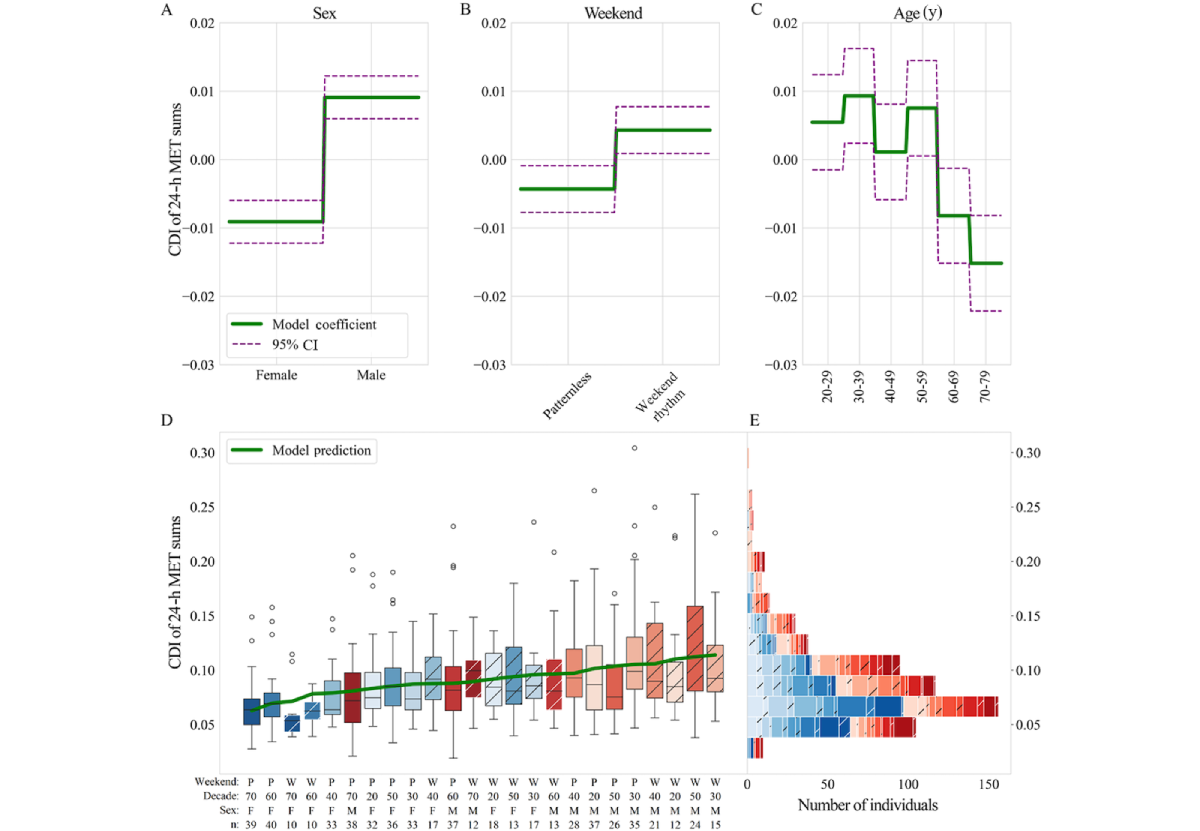
Generalized additive model–fitted factor functions for (A) sex, (B) weekend rhythm (WR), and (C) age with CIs. (D) Box plot of consecutive disparity index (CDI) of 24-hour metabolic equivalent of task (MET) sums for each unique phenotype group in order of the model prediction (green line) for that phenotype group. Weekend-Age-sex categories (P: patternless, W: weekend rhythm, Decade: age by decade, eg “70” is individuals in their 70s; F: Female individuals; M: Male individuals) are colored as in Figure 4A, and hatching indicates the presence of a WR in individuals in the labeled group. (E) Stacked histogram (ordered for visual clarity) of the number of individuals in CDI bins labeled by phenotype group, highlighting the overlap of each group in most bins.

The CDI was developed to improve upon the PV by accounting for the chronological order of measurements in a time series [37]. The CDI describes time series variability through the average rate of change between consecutive values:


CDI = (1 / (n-1)) Σ^n-1^_i=1_ |ln(p_i+1_ / p_i_)| **(5)**

where n is the length of the time series and p_i_ is the value in the series at time i [37] (Figures 2C and 2D, 3D, 4A and 4B, and 5A-5E).

### Analysis of PA by Cyclic Status

Every participant’s cyclic status (the label *cyclic* describes the presence of an approximately 28-day temperature rhythm generated by menstrual cycles) was determined through methods described in the study by Bruce et al [15]. Briefly, autocorrelation profiles were generated from nightly maximum temperature recordings (not shown). Only cyclic individuals’ temperature trend deviation autocorrelation signals show a wave-like structure. Profiles were classified as cyclic or acyclic by hierarchical clustering of pair-wise distances between signals (pair-wise distances calculated with dynamic time warping; not shown). Of the 298 female participants in this cohort, hierarchical clustering classified 193 (64.8%) as acyclic and 105 (35.2%) as cyclic; moreover, 297 (99.7%) of the 298 male participants were classified as acyclic. The temperature trend deviation autocorrelation signal for the male participant classified as cyclic did not show a wave-like structure; therefore, the male participant was manually reclassified as acyclic. Of the 105 female participants classified as cyclic, 102 (97.1%) were aged between 20 and 49 years, and 3 (2.9%) were aged between 50 and 59 years. Of the 193 female participants classified as acyclic, 48 (24.9%) were aged <50 years and 145 (75.1%) were aged >= 50 years.

Analysis of PA by cyclic status focused on the CDI variability metric and daily 24-hour MET sum metric. We chose 24-hour MET sums for analysis to focus on the overall variability due to PA in contrast to asleep or awake sums that vary with time spent in the state, as described in the Cohort and MET Data Foundational Analysis subsection. The CDI variability metric was chosen due to its accounting for chronological order, as described in the Variability Metrics of MET Sums subsection.

The autocorrelation and clustering techniques used to classify participants as cyclic or acyclic were also used to determine whether cyclic individuals had unique structures in daily 24-hour MET sums, such as a 28-day structure.

The means and CDIs of 24-hour MET sums were calculated for each individual over all 206 days present in the data and compared across cyclic status (cyclic female individuals vs all acyclic individuals of either sex; Kruskal-Wallis test). The CDIs of 24-hour MET sums were also compared across groups of individuals with unique combinations of sex and cyclic status (acyclic male individual, cyclic female individual, and acyclic female individual; Kruskal-Wallis test with Bonferroni correction for 3 comparisons and post hoc Dunn test [Figure 2D]). Cyclic and acyclic female individuals of the same age were compared to control for the uneven age distributions between the 2 groups (cyclic female individuals aged 20-59 y vs acyclic female individuals aged 20-59 y and cyclic female individuals aged 20-49 y vs acyclic female individuals aged 20-49 y; Kruskal-Wallis test). The effect of cyclic female individuals on the variability of the whole female population was calculated using IQR distributions, as described in the Statistical Methods subsection.

### Analysis by Weekend Rhythm in PA

Analysis by weekend rhythm in PA focused on the CDI variability metric and daily 24-hour MET sum metric. We chose 24-hour MET sums for analysis to focus on the overall variability due to PA in contrast to asleep or awake sums that vary with time spent in the state, as described in the Cohort and MET Data Foundational Analysis subsection. The CDI variability metric was chosen due to its accounting for chronological order, as described in the Variability Metrics of MET Sums subsection.

To determine whether PA rhythms existed on a weekly timescale, we examined a hierarchically clustered heat map (*seaborn* Python library: *seaborn.clustermap*, version 0.12.2 [36]) of unfilled and intraindividual *z* scored 24-hour MET sum data (not shown). Hierarchical clustering of unfilled (nonimputed) data ensured that clustered structures were not artifacts of data filling (eg, the median week imputation in the phase-dependent filling method may introduce weekly rhythms), and *z* scoring highlighted groups with similar patterns of change regardless of their baseline PA. Hierarchical clustering was performed on 4 consecutive months of data. The same 4 months were chosen for every individual to avoid days with larger proportions of missing data at the beginning and end of the study period. We observed 2 groups with different weekly PA rhythms on the heat map: 1 group with high 24-hour MET sums on weekends relative to their own weekday MET sums and 1 group with low 24-hour MET sums on weekends relative to their own weekday MET sums. These rhythms were defined as weekend rhythms, where the group with relatively high 24-hour MET sums on weekends was further identified as the weekend high PA rhythm group, and the second group was identified as the weekend low PA rhythm group.

Convinced that weekend rhythms were not artifacts of data filling, we performed agglomerative clustering on filled MET data (filling methods are described in the Data Filling subsection) to identify individuals with weekend high and weekend low PA rhythms. Agglomerative clustering was performed on 4 consecutive months (the same months used in the hierarchical clustering) of the filled and intraindividual *z* scored 24-hour MET sum data using the *scikit-learn* Python package (*sklearn.cluster.AgglomerativeClustering*, version 1.1.3 [41]). Clustering into 5 groups (Figure 3A) allowed for the identification of both the weekend high PA rhythm group (Figure 3B) and the weekend low PA rhythm group (Figure 3C), hereinafter referred to as the weekend high cluster and the weekend low cluster.

To confirm the presence of the weekend rhythms observed on the heat map (Figures 3A-C, top), we calculated the average 24-hour MET sum for each day in the consecutive 4 months across all participants (Figure 3A, bottom), across only participants in the weekend high cluster (Figure 3B, bottom), and across only participants in the weekend low cluster (Figure 3C, bottom). These averages were visualized as a line plot with the mean across all days in that group layered on top (Figures 3A-C, bottom).

To assess the differences between individuals with different weekend rhythms and those without weekend rhythms (patternless), the mean and CDI of 24-hour MET sums were calculated for each individual over the 4 consecutive months used to cluster the individuals by PA rhythm. The means were compared across weekend high, weekend low, and patternless clusters (Kruskal-Wallis test, Bonferroni correction for 3 comparisons, and post hoc Dunn test) while the CDIs were only compared across weekend rhythm (the aggregated group of individuals with either weekend high or weekend low PA rhythm) and patternless clusters (Kruskal-Wallis test between 2 groups). The CDIs were only compared across the presence or absence of a weekend rhythm because the direction of change in 24-hour MET sums on the weekend does not affect the CDI.

The CDI of 24-hour MET sums were also compared across groups of individuals with unique combinations of sex and PA rhythm (male individuals with weekend patterns, female individuals with weekend patterns, patternless male individuals, and patternless female individuals; Kruskal-Wallis test, Bonferroni correction for 6 comparisons, and post hoc Dunn test; Figure 3D). The effect of weekend rhythms on the variability of the whole male and female population was calculated using IQR distributions as described in the Statistical Methods subsection.

### Analysis of PA by Age

Analysis of PA by age focused on the CDI variability metric and daily 24-hour MET sum metric. We chose 24-hour MET sums for analysis to focus on the overall variability due to PA in contrast to asleep or awake sums that vary with time spent in the state, as described in the Cohort and MET Data Foundational Analysis subsection. The CDI variability metric was chosen due to its accounting for chronological order, as described in the Variability Metrics of MET Sums subsection.

The means and CDIs of 24-hour MET sums were calculated for each individual over all 206 days and compared across age categories (Kruskal-Wallis test, Bonferroni correction for 15 comparisons, and post hoc Dunn test). The CDIs of 24-hour MET sums were also compared across sex groups in the same age category (Kruskal-Wallis test, Bonferroni correction for 6 comparisons, 6 age groups; and post hoc Dunn test; Figure 4A) and across age categories within the same sex group (Kruskal-Wallis test, Bonferroni correction for 15 comparisons, and post hoc Dunn test; Figure 4B). A boxen plot (*seaborn* Python library: *seaborn.boxenplot*, version 0.12.2 [36]), also known as a letter-value plot, was used to visually compare male and female individuals within age groups (Figure 4A). A boxen plot is similar to a box plot but represents the whiskers as a variable number of quantiles. If the quantiles are sufficiently unique, meaning that they do not include values from other quantiles, they are represented as a box. This leaves 5 to 8 outliers on each side.

The effect of each age group on the variability of the whole male or female population was calculated using IQR distributions as described in the Statistical Methods subsection.

### Generalized Additive Model of the Features Found to Have Significant Impact on 24-Hour MET Sum CDIs Across Individuals: Sex, Age, and Weekend Rhythm

Previous studies have used generalized additive models (GAMs) to predict health outcomes using sex and age as features [42,43]. In this study, a GAM was used to rank the effect of variables on 24-hour MET sum CDIs and detect groups with outlier intraindividual variability (Figures 5A-E). A GAM was built in Python using the package *pyGAM* (*pygam.LinearGAM*, version 0.9.1 [44]).

Three initial models were tested: a model with an identity link and a factor term for all variables analyzed in this paper (sex, age, weekend rhythm, and cyclic status), all variables and all 2-way interactions (sex-age, age–weekend rhythm, etc), and all variables with all 2-way and all 3-way interactions (sex-age–cyclic status, etc). Model performance was assessed using the likelihood ratio pseudo-*R*^2^ metric, which represents the proportional reduction in the deviance and was reported as a percentage. The final model does not include cyclic status because its effects were not significant (refer to the Results section); thus, the factor terms were fit to sex, age, and weekend rhythm categories (sex: female or male; age: 20-29, 30-39, 40-49, 50-59, 60-69, or 70-79 y; weekend rhythm: weekend rhythm or patternless; Figures 5A-C). This resulted in the following GAM structure:


G(E(CDI)) = β_0_ + f_sex_(sex) + f_WR_(WR) + f_age_(age) **(6)**

where g is an identity link function, E(CDI) denotes the expected CDI value, β_0_ is the intercept of the model, and WR represents weekend rhythm. Individual feature importance was determined by the magnitude of the coefficients in each level of the factor terms and by the change in null deviance when each feature was left out.

### Ethical Considerations

The University of California San Francisco (UCSF) Institutional Review Board (IRB, IRB# 20-30408) and the U.S. DOD Human Research Protections Office (HRPO, HRPO# E01877.1a) approved of all study activities, and all research was performed in accordance with relevant guidelines and regulations and the Declaration of Helsinki. All participants provided informed electronic consent. We did not pay participants for participation and all participant data were de-identified by Oura prior to data transfer.

## Results

### Cohort and MET Data Foundational Analysis

As an initial comparison of MET values between the sexes, we visually assessed minute-level MET value time series and distributions for 2 representative individuals (Figures 1A and B). We observed a variation in MET values between awake and asleep states, with increased MET values during awake time periods, as expected (Figures 1A and B, left). In addition, we found that the distribution of MET values seemed highly dependent on asleep or awake state (Figures 1A and B, right); therefore, further comparisons used daily aggregated MET values separated into sums over 24 hours, only awake time periods, or only asleep time periods. The distributions of mean 24-hour, awake, and asleep daily MET sums for female and male individuals over the 206 days overall were not significantly different (Table 1; Figures 1C and D). However, we observed an apparent increase in the mean of 24-hour MET sums for male individuals at the upper extreme (Figure 1C). Consistent with this observation, a comparison of the individuals’ mean of 24-hour MET values (Figure 1C, right) revealed that the 60 male individuals with the largest average 24-hour MET sum had a significantly higher average than the top 60 female individuals (Kruskal-Wallis H=10.25; *P*=.001; Cohen *d_m_*=0.34). We also observed differences between male and female intraindividual variability: male individuals had significantly larger SDs than female individuals for both awake and 24-hour MET sums (Table 1; Figure 1E).

**Table 1:** Mean and SD statistics by time state: Kruskal-Wallis test across the sexes for the mean and SD of each time state (Bonferroni-corrected significance threshold for 3 comparisons: *P*=.02).

Statistic and MET^a^ sum		Kruskal-Wallis H statistic	*P* value	Sex with larger median
**Mean**				
	24 h	0.36	.55	Male
	Awake	0.40	.53	Male
	Asleep	1.01	.31	Female
**SD**				
	24 h	38.54	<.001	Male
	Awake	11.60	<.001	Male
	Asleep	0.03	.85	Female

^a^MET: metabolic equivalent of task.

### Variability Metrics of MET Sums

In total, 4 intraindividual variability metrics were calculated: SD, CV, PV, and CDI. The most appropriate metric of variability for our analyses was the CDI because of its accounting for chronological order and nondependence on the mean for calculation. Other metrics were included as controls to validate the statistical findings from CDI analyses. Further analyses used only the CDI as a variability metric.

The CV and PV of male individuals were significantly larger than those of female individuals for awake and 24-hour MET sums (Figures 2A and B; Table 2), while the CDI for 24-hour MET sums was significantly larger for male individuals than for female individuals (Figure 2C; Table 2; Cohen *d_m_*=0.35). In all 3 metrics, asleep MET sum intraindividual variability was not significantly different across the sexes (Figure 1D; Table 1; Figures 2A and C; Table 2).

**Table 2:** Variability metrics by time state: Kruskal-Wallis test across the sexes for coefficient of variation (CV), proportional variability index (PV), and consecutive disparity index (CDI) of each time state (Bonferroni-corrected significance threshold for 3 comparisons: *P*=.02).

Statistic and MET^a^ sum		Kruskal-Wallis H statistic	*P* value	Sex with larger median
**CV**				
	24 h	43.70	<.001	Male
	Awake	9.36	.002	Male
	Asleep	0.17	.68	Male
**PV**				
	24 h	37.90	<.001	Male
	Awake	10.97	<.001	Male
	Asleep	3.12	.08	Male
**CDI**				
	24 h	29.51	<.001	Male
	Awake	0.90	.34	Male
	Asleep	0.96	.33	Male

^a^MET: metabolic equivalent of task.

### Analysis of PA by Cyclic Status

Neither 28-day (or near 28-d) temporal structures nor any unique temporal structure in daily 24-hour MET sums were identified in cyclic individuals. Cyclic female participants and all acyclic participants (male or female) did not have significantly different mean 24-hour MET sums (Kruskal-Wallis H=0.46; *P*=.50; data not shown) or significantly different 24-hour MET sum CDIs (Kruskal-Wallis H=1.03; *P*=.31; Figure 2D). However, we found a significant difference between the CDI values of 24-hour MET sums for male participants, cyclic female participants, and acyclic female participants (Kruskal-Wallis H=32.36; *P*<.001; Figure 2D). A Dunn test revealed that female participants exhibited lower intraindividual variability than male participants, regardless of cyclic status (male participants vs cyclic female participants: *P*=.006; Cohen *d_m_*=0.27; male participants vs acyclic female participants: *P*<.001; Cohen *d_m_*=0.41), and that cyclic female participants and acyclic female participants were not significantly different (*P*=.09). Cyclic female participants and acyclic female participants of the same age were also compared to confirm that the uneven age distribution between the 2 groups did not contribute to there being no statistical difference between the groups (Kruskal-Wallis test, cyclic female participants aged 20-59 y [n=105] vs acyclic female participants aged 20-59 y [n=94]: H=2.30; *P*=.13; cyclic female participants aged 20-49 y [n=102] vs acyclic female participants aged 20-49 y [n=48]: H=0.53; *P*=.47). We then compared the population variability of the whole female population and the female population excluding cyclic female participants, as described in the Effect of Subpopulations subsection. Removing cyclic female participants from the female population did not significantly reduce the whole female population variability 24-hour MET sums (Kruskal-Wallis H=0.12; *P*=.73).

### Analysis by Weekend Rhythm in PA

Agglomerative clustering of 4 months of data per individual across the whole cohort revealed clusters of individuals sharing prominent PA rhythms on a weekly timescale (Figure 3A). Two clusters of individuals with weekend rhythms were identified: a “weekend high” cluster (labeled the “weekend high PA rhythm group” in dark green in Figures 3A and B) and a “weekend low” cluster (labeled the “weekend low PA rhythm group” in purple in Figures 3A and C). The 3 clusters without weekend rhythms are referred to as “patternless” clusters (labeled orange, pink, and light green in Figure 3A).

Significant differences in the means of 24-hour MET sums existed between individuals in the weekend high cluster, weekend low cluster, and the patternless clusters (Kruskal-Wallis H=9.18; *P*=.01; Bonferroni-corrected significance threshold: *P*=.02; data not shown). The weekend high cluster had significantly larger mean 24-hour MET sums than the weekend low cluster and the patternless clusters (Dunn test, weekend high vs weekend low: *P*=.007; weekend high vs patternless: *P*=.01). Cohen *d_m_* effect sizes between significantly different groups were 0.41 (weekend high vs weekend low) and 0.22 (weekend high vs patternless).

Next, we grouped the individuals with any weekend rhythm (weekend high or weekend low) to examine intraindividual variability. The cluster of individuals with either weekend rhythm had significantly larger 24-hour MET sum CDIs than individuals in the patternless clusters (Kruskal-Wallis H=10.13; *P*=.001; Cohen *d_m_*=0.20; data not shown). The Cohen *d_m_* effect size between the CDIs of 24-hour MET sums for male and female individuals was 0.35, suggesting that sex explained more intraindividual variability than PA rhythms on the weekly timescale.

We found significant effects of sex and weekend rhythm on 24-hour MET sum CDIs (Kruskal-Wallis test, Bonferroni-corrected significance threshold: *P*=.008; H=34.60; *P*<.001; Figure 3D). Male individuals had larger 24-hour MET sum CDIs than female individuals in the same cluster (Dunn test, patternless cluster: *P*<.001; Cohen *d_m_*=0.32; weekend rhythm cluster: *P*=.003; Cohen *d_m_*=0.51). In addition, male individuals in the weekend rhythm cluster had significantly larger 24-hour MET sum CDIs than female individuals from the patternless clusters (Dunn test, *P*<.001; Cohen *d_m_*=0.49); however, female individuals in the weekend rhythm cluster did not have significantly larger 24-hour MET sum CDIs than male individuals in the patternless clusters (Dunn test: *P*=.24). We found no significant effect between clusters within sex on 24-hour MET sum CDIs: male individuals in the weekend rhythm cluster did not differ from those in the patternless clusters (Dunn test, *P*=.02), and nor did female individuals in the weekend rhythm cluster differ from those in the patternless clusters (Dunn test, *P*=.06).

We compared the variability of the whole male and female populations to the populations excluding individuals with weekend rhythms using the strategy described in the Effect of Subpopulations subsection. Excluding individuals with weekend rhythms did not reduce the population variability of 24-hour MET sums of either the whole male or female population (Kruskal-Wallis test, Bonferroni-corrected significance threshold: *P*=.025; all female individuals vs female individuals without weekend rhythm clusters: H=2.62; *P*=.11; all male individuals vs male individuals without weekend rhythm clusters: H=4.46; *P*=.03).

### Analysis of PA by Age

We found significant differences in mean 24-hour MET sums across age groups (Kruskal-Wallis H=24.30; *P*<.001; Bonferroni-corrected significance threshold for 15 comparisons: *P*=.003; data not shown). Individuals aged 70 to 79 years had significantly smaller mean 24-hour daily MET sums than those aged 30 to 39 and 50 to 59 years (Dunn test, 70-79 y vs 30-39 y: *P*<.001; Cohen *d_m_*=0.54; 70-79 y vs 50-59 y: *P*<.001; Cohen *d_m_*=0.39), and individuals aged 60 to 69 years had significantly smaller mean 24-hour daily MET sums than those aged 30 to 39 years (Dunn test, 60-69 y vs 30-39 y: *P*=.003; Cohen *d_m_*=0.28). Other comparisons of mean 24-hour MET sums between age groups were not statistically significant (data not shown).

Differences in 24-hour MET sum CDIs existed across age groups (Kruskal-Wallis H=40.55; *P*<.001; Bonferroni-corrected significance threshold for 15 comparisons: *P*=.003; Table 3). Individuals aged 70 to 79 years had significantly smaller 24-hour MET sum CDIs than those aged 20 to 29, 30 to 39, 40 to 49, and 50 to 59 years (Table 3). Individuals aged 60 to 69 years had significantly smaller 24-hour MET sum CDIs than those aged 30 to 39 and 50 to 59 years (Table 3). The Cohen *d_m_* effect sizes between the groups that were significantly different ranged from 0.36 to 0.56, suggesting that age explained more intraindividual variability than sex (Cohen *d_m_*=0.35) and weekly rhythm (Cohen *d_m_*=0.20).

Having found a significant effect of sex and age bin, we carried out pair-wise comparisons of sex within each age bin and found that male individuals aged 30 to 39 years and 40 to 49 years had significantly higher 24-hour MET sum CDIs than female individuals in the same age groups (Kruskal-Wallis test, Bonferroni-corrected significance threshold for 6 comparisons: *P*=.008; male individuals aged 30-39 y vs female individuals aged 30-39 y: H=8.62; *P*=.003; Cohen *d_m_*=0.37; male individuals aged 40-49 y vs female individuals aged 40-49 y: H=8.64; *P*=.003; Cohen *d_m_*=0.33; Figure 4A). We further note that while the remaining comparisons were not significant, the trend in every age group was toward the same direction of difference, with male individuals having higher median CDI at all ages (Kruskal-Wallis test, Bonferroni-corrected significance threshold for 6 comparisons: *P*=.008; male individuals aged 20-29 y vs female individuals aged 20-29 y: H=0.96; *P*=.33; male individuals aged 50-59 y vs female individuals aged 50-59 y: H=0.78; *P*=.38; male individuals aged 60-69 y vs female individuals aged 60-69 y: H=6.58; *P*=.01; male individuals aged 70-79 y vs female individuals aged 70-79 y: H=6.38; *P*=.01; Figure 4A).

Female individuals aged 70 to 79 years were significantly less variable than those aged 20 to 29, 30 to 39, and 50 to 59 years; and female individuals aged 60 to 69 years were significantly less variable than those aged 50 to 59 years (Figure 4B; Table 4). Cohen *d_m_* effect sizes for these differences were between 0.50 and 0.69 (Table 4). Male individuals aged 70 to 79 years were significantly less variable than those aged 30 to 39 years, with a Cohen *d_m_* effect size of 0.40 (Figure 4B; Table 5).

We compared the variability of the whole male and female populations excluding each single age group using the strategy described in the Effects of Subpopulations subsection. The IQR distributions composed of the daily IQRs of population 24-hour MET sums were not significantly different between (1) the whole population and (2) the population without any single age group, except in 1 comparison (Table 6). The whole female population and the female population without individuals aged 60 to 69 years had significantly different IQRs of 24-hour MET sums such that the female population variability was increased by the presence of female individuals aged 60 to 69 years (Table 6; Cohen *d_m_=*0.18). Using the rule formulated by Lehr [35], we calculated the effect of the increased population variability caused by female individuals aged 60 to 69 years on the approximate required sample size to detect a statistically significant difference. We found that to detect a difference of 40 (approximately the difference in 24-hour MET sums resulting from a 20-minute walk), the exclusion of female individuals aged 60 to 69 years results in a sample size reduction from 1088 to 1047 (a reduction of 3.8%). For a difference of 100 (approximately the difference in 24-hour MET sums resulting from 20 minutes of moderate-intensity activity), the exclusion results in a sample size reduction from 174 to 167 (a reduction of 4%); and for a difference of 180 (approximately the difference in 24-hour MET sums resulting from 20 minutes of high-intensity activity), the exclusion results in a sample size reduction from 54 to 52 (a reduction of 3.7%).

**Table 3:** Age bin statistics. The diagonal shows the median consecutive disparity index for each age bin. Below and to the left of the diagonal are the *P* values from the post hoc Dunn tests comparing each age group (significant comparisons are italicized). Above and to the right of the diagonal are the modified Cohen *d* effect sizes of the comparisons that were significantly different (Kruskal-Wallis test, Bonferroni-corrected significance threshold for 15 comparisons: *P*=.003).

	20-29 y	30-39 y	40-49 y	50-59 y	60-69 y	70-79 y
20-29 y	0.082	—^a^	—	—	—	0.38
30-39 y	.38	0.087	—	—	0.47	0.56
40-49 y	.68	.20	0.081	—	—	0.36
50-59 y	.64	.67	.38	0.089	0.43	0.50
60-69 y	.005	*<.001* ^b^	.02	*.001*	0.07	—
70-79 y	*<.001*	*<.001*	*<.001*	*<.001*	.18	0.065

^a^Not applicable.

^b^Italicized values indicate significance.

**Table 4:** Age bin statistics for female individuals. The diagonal shows the median consecutive disparity index for each age bin. Below and to the left of the diagonal are the *P* values from the post hoc Dunn tests comparing each age group (significant comparisons are italicized). Above and to the right of the diagonal are the modified Cohen *d* effect sizes of the comparisons that were significantly different (Kruskal-Wallis test, Bonferroni-corrected significance threshold for 30 comparisons: *P*=.002).

	20-29 y	30-39 y	40-49 y	50-59 y	60-69 y	70-79 y
20-29 y	0.082	—^a^	—	—	—	0.60
30-39 y	.88	0.080	—	—	—	0.64
40-49 y	.17	.22	0.074	—	—	—
50-59 y	.77	.66	.10	0.084	0.50	0.69
60-69 y	.003	.004	.10	*.001* ^b^	0.068	—
70-79 y	*<.001*	*<.001*	.005	*<.001*	.22	0.062

^a^Not applicable.

^b^Italicized values indicate significance.

**Table 5:** Age bin statistics for male individuals. The diagonal shows the median consecutive disparity index for each age bin. Below and to the left of the diagonal are the *P* values from the post hoc Dunn test comparing each age group (significant comparisons are italicized). Above and to the right of the diagonal is the modified Cohen *d* effect size of the comparison that was significantly different (Kruskal-Wallis test, Bonferroni-corrected significance threshold for 30 comparisons: *P*=.002).

	20-29 y	30-39 y	40-49 y	50-59 y	60-69 y	70-79 y
20-29 y	0.087	—^a^	—	—	—	—
30-39 y	.18	0.096	—	—	—	0.40
40-49 y	.56	.45	0.091	—	—	—
50-59 y	.72	.32	.81	0.100	—	—
60-69 y	.29	.02	.10	.16	0.081	—
70-79 y	.05	*<.001* ^b^	.01	.02	.36	0.076

^a^Not applicable.

^b^Italicized values indicate significance.

**Table 6:** Kruskal-Wallis test of daily IQRs (n=206) between the whole female or male population and the whole female or male population with 1 age group removed (Bonferroni-corrected significance threshold for 12 comparisons: *P*=.004).

Sex and removed age group (y)		Kruskal-Wallis H statistic	*P* value
**Male**			
	20-29	0.32	.57
	30-39	0.33	.57
	40-49	3.57	.06
	50-59	0.10	.75
	60-69	4.75	.03
	70-79	7.40	.007
**Female**			
	20-29	0.17	.68
	30-39	1.65	.20
	40-49	4.81	.03
	50-59	2.89	.09
	60-69	11.11	<.001
	70-79	7.17	.007

### GAM of the Features Found to Have Significant Impact on 24-Hour MET Sum CDIs Across Individuals: Sex, Age, and Weekend Rhythm

A GAM was used to summarize the contributions of sex, age, cyclic status, and weekend rhythm to 24-hour MET sum CDIs across individuals. Three initial models were tested to find the best model for explaining population variability in CDI while retaining interpretability: (1) a model with an identity link and a factor term for all variables analyzed in this paper (sex, age, weekend rhythm, and cyclic status), (2) all variables and all 2-way interactions (sex-age, age–weekend rhythm, etc), and (3) all variables with all 2-way and all 3-way interactions (sex-age–cyclic status, etc). The first model explained 11.5% of the null deviance, but the cyclic status term was not significantly different from 0 (*P*=.17). The last 2 models explained 1.6% and 2.8% more of the null deviance than the first model, where again cyclic status was not significant (second model: *P*=.63; third model: *P*=.84). These analyses support our finding that acyclic and cyclic individuals did not have significantly different CDI values. Given the marginal increase in null deviance explained for the substantial increase in model complexity (6 and 10 additional relational features in the second and third models, respectively) and the increased difficulty of interpreting the models with multiple interaction terms (4 terms in the first model vs 10 and 14 in the second and third models, respectively), the first model was chosen for further analysis. To construct the final model, the cyclic status variable was removed from the first model because the term was not significantly different from 0, leaving the final variables as sex, age, and weekend rhythm.

Unique combinations of the categories (physiological phenotypes) across the final variables resulted in 24 phenotype groups (eg, female, 20-29 y, and weekend rhythm) for which the model predicted a CDI value. Each of the variables had a significant effect on the model prediction (sex: *P*<.001; weekend rhythm: *P*=.01; and age: *P*<.001). The null deviance explained by the final model decreased by 4.9% when sex was excluded as a feature, by 4.7% when age was excluded as a feature, and by 0.92% when weekend rhythm was excluded as a feature, indicating that sex and age were the most important features in this model for predicting CDI. Coefficient magnitudes indicated that sex and specific age bins had the greatest effect on CDI out of these categories: sex (Figure 5A) had an overall effect of −0.0091 for female individuals 0.0091 for male individuals, weekend rhythm (Figure 5B) had an overall effect of −0.0043 for patternless individuals and 0.0043 for those with weekend rhythms, and age bin (Figure 5C) had an overall effect of −0.015 to 0.0093 (20-29 y: 0.0055, 30-39 y: 0.0093, 40-49 y: 0.0011, 50-59 y: 0.0075, 60-69 y: −0.0082, and 70-79 y: −0.015). However, the overall deviance explained by the final model was 11.3%, indicating a low proportion of null deviance explained by the model. This is consistent with our Cohen *d_m_* analyses that found the difference in median CDI between categories to be smaller than the size of the IQRs of the categories themselves (refer to our discussion of sex, weekly rhythms, and age in the Variability Metrics of MET Sums, Analysis of Weekend Rhythm in PA, and Analysis of PA by Age sections; Cohen *d_m_*=0.35, 0.20, and 0.36-0.56, respectively). Together, both these analyses indicated that even timescales of change that were significant sources of variability in CDI were not substantial sources of variability that would likely weaken statistical power. GAM analysis further showed that the intersection of sex with specific age bins (30-39 y, 50-59 y, 60-69 y, and 70-79 y) had the greatest impact on GAM predictions. However, it also confirmed that no single category was in itself a substantial source of variability in the population. Model predictions did not align with unique values for each phenotype group, and there was significant overlap between the groups in CDI range (Figures 5D and E).

## Discussion

### Principal Findings

In this work, we found evidence to reject the hypothesis that it is necessary to exclude women as research participants when assessing PA-related behaviors. Sex and cyclic status were found to represent different populations, and neither sex nor menstrual cycles substantially increased the intraindividual variability of PA. Rather, we found that female individuals exhibit significantly less intraindividual variability than male individuals, regardless of their cyclic status. This study also demonstrates that the exclusion of either sex is unwarranted because the overall difference in intraindividual PA variability was small. However, this work did reinforce the utility of SABV because we found differences by sex in the contributions of different timescales (weekends and age) to the patterns of change in PA over time.

Male and female individuals showed no significant differences between mean 24-hour MET sums, but the 60 most active male individuals were significantly more active than the 60 most active female individuals. The SD, CV, PV, and CDI values of 24-hour MET sums were all significantly different by sex. As the CDI captures local changes instead of only global structure, we deemed the CDI the best indicator of continuous intraindividual variability for time series data. Cyclic status had no effect on 24-hour MET sum CDIs, and no temporal structures on the timescales of menstrual cycles were found in cyclic individuals (ie, the approximately 28-day rhythms in these individuals’ temperature data [15] were not reflected in their PA).

We did find that some participants in the dataset had temporal structure on the timescales of weeks. Participants with weekend rhythms were found to have higher intraindividual variability (24-hour MET sum CDI) than those without weekend rhythms (patternless), regardless of sex. However, within each sex, participants with weekend rhythms did not have significantly different intraindividual variability compared to those without weekend rhythms, nor did their inclusion increase the population variability of the whole population of male or female individuals. Male individuals were more intraindividually variable than female individuals, regardless of weekend rhythm. Without an SABV analysis, we may have concluded that the CDI was significantly different between individuals with weekend patterns and those without when the actual cause of this deviation seems to be due to the fact that male PA is more variable within individuals than female PA.

We also found that sex differences existed in the presence of weekend rhythms. Interestingly, those with weekend effects were more likely to be male, although both sexes were represented in this category (182 individuals had weekend rhythms, n=85, 46.7% were female individuals and n=97, 53.3% were male individuals). This may be because weekends play a large role in modulating behavior; for example, work schedules may inhibit PA during weekdays, leading some individuals to make up their PA debt on weekends. Others may have active work schedules and seek to rest and recuperate on weekends. One study found that individuals who were more active on weekdays than on weekends had lower education and were more likely to work manual occupations than those who were consistently inactive [45]. A higher group membership of male individuals (female individuals: 55/133, 41.4%; male individuals: 78/133, 58.6%) in the weekend high group may also support the finding that female individuals have higher rates of inactivity [3] if increased activity on the weekend is due to participation in exercise.

Age did not have a consistent effect on intraindividual variability. When the data were sex disaggregated, female individuals aged 70 to 79 and 60 to 69 years were less variable than a few of the younger age groups; however, among male individuals, only 1 difference was observed: male individuals aged 70 to 79 years were less variable than those aged 30 to 39 years. This decrease in intraindividual variability in the oldest age groups is likely caused by increased sedentary behavior with increased age [46]. In addition, male individuals aged 40 to 49 and 30 to 39 years were more intraindividually variable than female individuals in the same age groups. This, again, is in contrast to the results when all individuals of both sexes were considered in statistical tests. If the data had not been sex disaggregated, we may have concluded that male intraindividual variability across age bins looks similar to female intraindividual variability when it evidently does not. The lack of difference across age bins in male individuals seems to be caused by increased population variability of 24-hour MET sum CDIs within each age bin when compared to female individuals. We note that female individuals aged 60 to 69 years were the only group to significantly increase the population variability of the whole female population. We used this group to test the hypothesis that excluding subgroups that significantly increased whole-population variability would meaningfully improve statistical power for the included groups. We found a change in sample size of <5% for computed comparisons. We argue that the benefits from reducing, for example, a 200-person study to a 192-person study are likely minimal compared to the value of including a whole other group so that the findings apply broadly to more people.

The effects of weekend rhythms and age, along with the lack of effects due to cyclic status, on intraindividual variability all suggest that sex alone is not an effective proxy for the presence of temporal structure or the intraindividual variability that may affect statistical analysis. In our final analysis, we used a multivariate (GAM) model that determined that while sex, weekend rhythm, and age have significant effects on intraindividual PA variability, only 11.3% of the population variability in 24-hour MET sum CDIs can be explained by these phenotypes. The analysis showed that age and sex had similar effects on intraindividual PA variability and that weekend rhythm had a much smaller effect comparatively. Cyclic status did not have a significant effect (consistent even in the more complex models) and in fact had less effect than any other timescale studied. The analysis also highlights the potential usefulness of intersectional phenotypes by showing that they provide more information about an individual than single phenotypes. Indeed, digital twinning is emerging as a computational approach for providing precision insights into health by grouping “similar” individuals (similar based on many potential features of their data) and then identifying signs or treatments specific to this group, as opposed to being limited to more classical demographics such as sex or ethnicity alone [47,48]. As these approaches mature, timescales of change such as menstrual cycles, weekend patterns, and circadian rhythms might prove to be useful features by which to define similarity. Even when the intraindividual variability is approximately equal across such groups (we found that only 11.3% of intraindividual variability can be accounted for by the various timescales in this work), the behaviors or needs of groups with different dynamics may still differ due to differing physiology.

Older female individuals with weekend rhythms seem to have the least intraindividual variability of all participant phenotypes (Figure 5D), perhaps indicating stronger behavioral routines in this phenotype group. Ironically, older female individuals, who are historically even more understudied than female individuals broadly [49,50], would seem to have mitigated concerns about increased intraindividual variability eroding statistical comparisons more than any other group, including the most historically overrepresented population of middle-aged male individuals. This is not an argument that men should be excluded—no group should be excluded from research, and no groups in our models exhibited an overwhelming amount of intraindividual variability that would reduce power in statistical comparison. Rather, this highlights that assumptions about who should be excluded in the interest of minimizing population variability and maximizing statistical power may have made statistical inference harder rather than easier (and may still be doing so when numerical examinations of these assumptions are absent in any given field of study). While the multivariate analysis suggests that, among the 4 variables studied, sex and age most affect intraindividual variability, none of these variables alone, nor their intersection, reliably predicted intraindividual variability. This suggests that no group is so different from the others as to warrant statistical exclusion.

The key assertion is that in the context of PA, which is the most commonly available longitudinal physiological measure for humans, we found no support for the hypothesis that female individuals broadly are more variable than male individuals.

### Limitations

This study aligns with our previous findings about the impacts of sex and menstrual cycles on variability in continuous temperature data [15]. As those analyses and the analyses presented here were conducted on the same cohort, it is possible that new cohorts would show different distributions. Additional studies would help identify the stability and context for variability in different phenotypes and populations; for example, we do not suggest that all older female individuals are less variable than all young male individuals—indeed, the least variable phenotype across the 3 characteristics of age, sex, and weekend rhythm had a substantially smaller sample size (Figure 5D) and therefore may well not be reliably representative of the broader population of older female individuals. Instead, we suggest that our longitudinal analyses found this to be the case in this modality (PA) in this dataset.

In addition, it is worth noting that MET is not equivalent to step count but rather an adjusted measure of activity, conditioned by the weight of the individual. While MET does not provide insights into total absolute activity or types of activity, it varies with activity intensity and thus provides a means of assessing different timescales of behavioral change across individuals’ data, as analyzed in this study. Although METs have been found to have systematic inaccuracies in energy expenditure estimates due to their reliance on body weight for calculation [51], this does not affect the relative change we analyzed in intraindividual variability. Furthermore, while the exact MET calculation used by Oura Ring is proprietary and not disclosed to us, Oura Ring (Gen 2) activity measurements displayed high correlation when validated against multiple accelerometers [30]. We encourage further study using different metrics to more fully describe the variability landscape from as many angles as might be relevant to other applications or fields of research.

### Comparison With Prior Work

This work joins a growing body of analyses that support the inclusion of both sexes in biomedical research [13,15-20,52-56]. The persistent sex bias in participant selection for biomedical research in humans and its detrimental impact on women’s health care has been thoroughly described previously [52-55]. The harmful exclusion of women and female individuals as participants has received increased attention in the past decade, including specific mention as a problem in the 2024 Presidential State of the Union Address [57]. Public attention to this issue, along with US [58] and international [59,60] policy changes affecting the inclusion of female individuals, has led to marked improvements in cohort equity [13,61]. However, many researchers still fail to include participants of both sexes in experiments; and those who do, often fail to perform SABV analyses [13,16,58]. Researchers’ resistance to include female individuals in both animal and human studies in biomedical research stems from the same concerns observed in sports and exercise medicine: including female individuals will increase intraindividual measurement variability due to hormone fluctuations and thus reduce statistical power [56]. Our results support the inclusion of female participants, consistent with many other studies that found that female participants do not reduce the statistical power of experiments due to substantial variability [16-20]. Both this work and our previous work on temperature variability found that sex does affect variability, but cyclic status alone does not account for the difference between male and female individuals [15]. Neither segregation by sex nor segregation by cyclic status alone seems to be a useful control for overall variability in these modalities [15]. As a result, our work suggests that exclusion for the sake of preserving statistical power is neither necessary nor justified.

While this study is related to sex bias in biomedical research at large, the findings presented here are most applicable and comparable to behavioral research (here considered a subset of biomedical research) and epidemiological research in PA because the variability metric used (the CDI of daily MET sums) approximates the amount of total exercise and movement in a day without consideration for the types of activity or physiological processes.

In regard to epidemiological research on PA, our findings did not reflect the general consensus that female individuals are less active than male individuals [3-5]. However, as discussed previously, METs have been found to have systematic inaccuracies in energy expenditure estimates [51] and may therefore inaccurately measure the amount of PA. Another potential cause for this discrepancy is that people who use wearables are more likely to be active than those who do not [62,63].

The effects of menstrual cycles on exercise performance have been studied previously, and the results are largely conflicting and inconclusive [9,10]. While this work does address PA variability in people with approximately 28-day temperature cycles, it differs from these studies in terms of metrics: these studies assess exercise performance metrics such as strength and endurance, while our analyses examine the intraindividual variability of a daily summary of behavior or PA. This study also does not examine specific stages of the menstrual cycle or exercise performance metrics; however, the absence of 28-day temporal patterns in 24-hour MET sums at least suggests that if menstrual cycle–related changes in exercise performance exist, they do not significantly affect behavior or the total amount of PA.

Instead of finding temporal structures on menstrual cycle timescales, we found temporal structures on weekly timescales, confirming the findings from other recent accelerometry studies that reported weekly rhythms in PA [45,64]. While this study did not use raw accelerometer data, it expands on previous studies in cohort age diversity [45] and the length of the study period [45,64]. However, these previous studies have focused on total amounts of activity rather than the presence of rhythms and are not directly comparable to this work. Weekend rhythms are not the main thrust of our work, but these findings may be of interest to those studying activity patterns.

### Conclusions

In conclusion, our findings support sex-based and age-based analyses in biomedical research involving PA, while rejecting the exclusion of female individuals, male individuals, weekend rhythm types, or any other specific intersectional phenotype from biomedical research based on the assumptions of increased intraindividual variability of PA interfering with statistical power.

## Appendix

Multimedia Appendix 1 Tables recording population SDs of each sex for each metabolic equivalent of task sum metric and for each sex subgroup for 24-hour metabolic equivalent of task sums, along with figures related to data filling. Population SDs are presented for their relevance to power analysis.

## Abbreviations

CDI: consecutive disparity index
CV: coefficient of variation
GAM: generalized additive model
HRPO: Human Research Protections Office
IRB: Institutional Review Board
MET: metabolic equivalent of task
PA: physical activity
PV: proportional variability index
SABV: sex as a biological variable
UCSF: University of California San Francisco

## Data Availability

Oura’s data use policy does not permit us to make wearable device data (collected via the Oura Ring) available to third parties. Access to anonymized and privacy-protected data may be granted to a qualified academic investigator upon completing agreements with Oura Health Oy and the investigators. Please contact Ashley E. Mason and Benjamin L. Smarr to obtain an application to obtain these data.
