# Supplementary material for: Sex Differences in the Variability of Physical Activity Measurements Across Multiple Timescales Recorded by a Wearable Device: Observational Retrospective Cohort Study
Source: J Med Internet Res. 2025 Apr 28;27:e66231. doi: 10.2196/66231 (PMC12070018; doi:10.2196/66231)
Supplement: Multimedia Appendix 1 [file jmir_v27i1e66231_app1.doc]

**Supplementary Table 1.** The standard deviation of the population distributions of mean 24-hour daily MET sum, awake daily MET sum, and asleep daily MET sum calculated from 206 days

| Population (n) | Population Standard Deviation of Individual Mean of MET Sums | | |
| --- | --- | --- | --- |
|  | 24-Hour | Awake | Asleep |
| Male (298) | 235 | 254 | 97.7 |
| Female (298) | 194 | 230 | 99.6 |

**Supplementary Table 2.** Population standard deviations of all subgroups studied were calculated from the population distributions of mean 24-hour daily MET sum. Weekly PA rhythm group individual means were calculated from 4 consecutive months of data and all other group’s individual means were calculated from all 206 days in the dataset.

| Population |  |  | Population Standard Deviation of Individual Mean of 24-hour MET Sums |
| --- | --- | --- | --- |
| Sex (n) |  |  |  |
| Female (298) |  |  | 194 |
| Weekly PA Rhythm (n) | |  |  |
|  | Patternless (213) |  | 212 |
|  | Weekend Rhythm (n) |  | 200 |
|  | Weekend High (55) | | 201 |
|  | Weekend Low (30) | | 187 |
| Cyclicity (n) | |  |  |
|  | Cyclic (105) |  | 175 |
|  | Acyclic (193) |  | 203 |
| Age (n) | |  |  |
|  | 20-29 (50) |  | 175 |
|  | 30-39 (50) |  | 172 |
|  | 40-49 (50) |  | 170 |
|  | 50-59 (49) |  | 219 |
|  | 60-69 (50) |  | 226 |
|  | 70-79 (49) |  | 177 |
| Male (298) |  |  | 235 |
| Weekly PA Rhythm (n) | |  |  |
|  | Patternless (201) |  | 240 |
|  | Weekend Rhythm (n) |  | 235 |
|  | Weekend High (78) | | 248 |
|  | Weekend Low (19) | | 150 |
| Cyclicity (n) | |  |  |
|  | Acyclic (298) |  | 235 |
| Age (n) | |  |  |
|  | 20-29 (49) |  | 278 |
|  | 30-39 (50) |  | 220 |
|  | 40-49 (49) |  | 221 |
|  | 50-59 (50) |  | 238 |
|  | 60-69 (50) |  | 247 |
|  | 70-79 (50) |  | 169 |


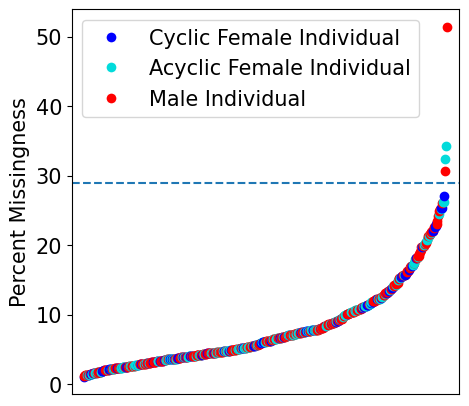


**Supplementary Figure 1.** Percent of values that were missing in each individual from the starting cohort sorted from least to greatest and labeled by their sex and cyclic status. Individuals above the horizontal dashed line at 29% were removed from the cohort for this analysis, leaving 596 total individuals with less than 29% total missingness in their data.


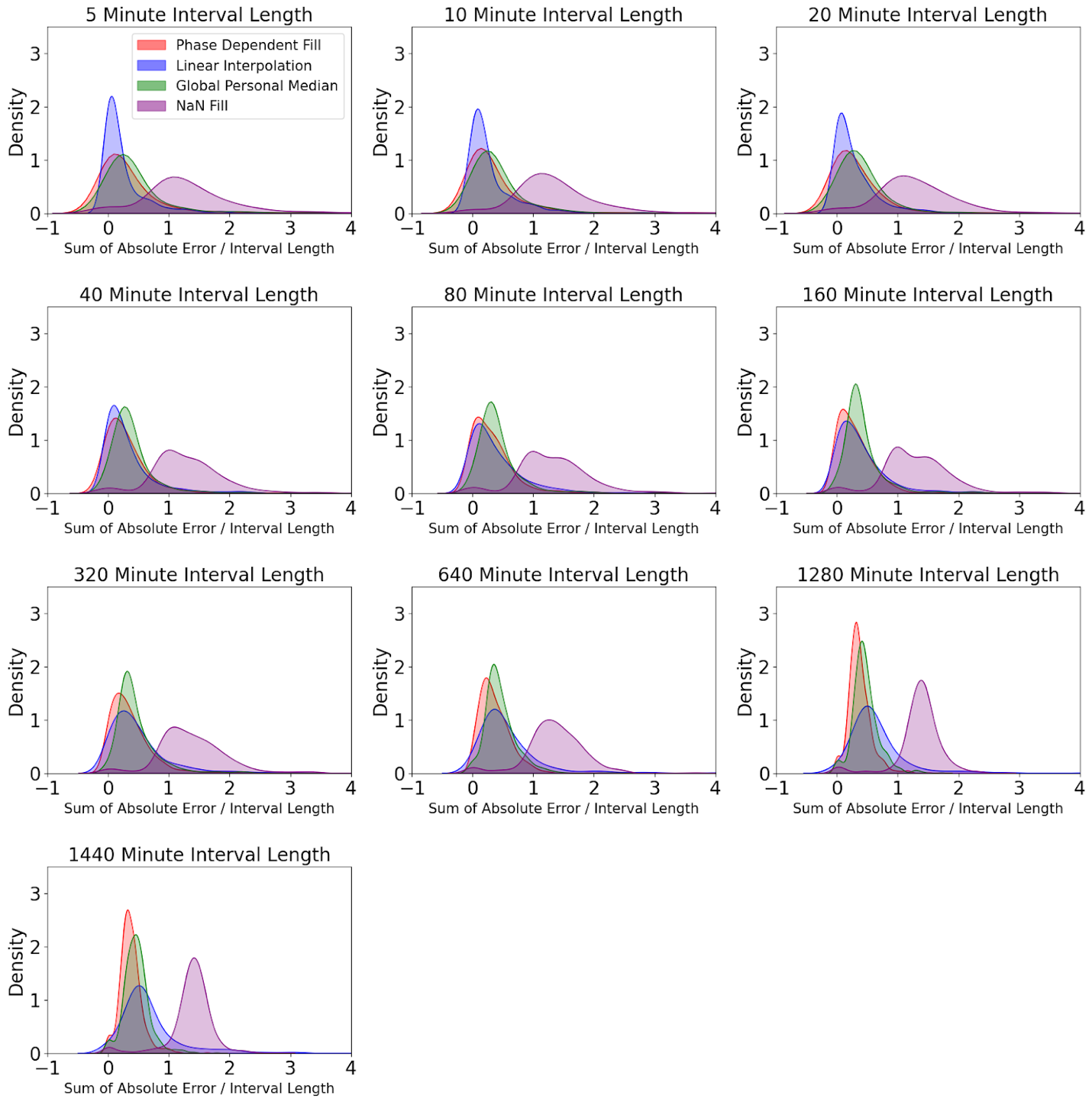
**Supplementary Figure 2.** Kernel density estimate plots of the error generated by each filling method on simulated missing MET data. Error is shown as the sum of the absolute error divided by the interval length, or average error per minute. Each distribution is composed of exactly 596 measurements, one of each interval length for every individual.


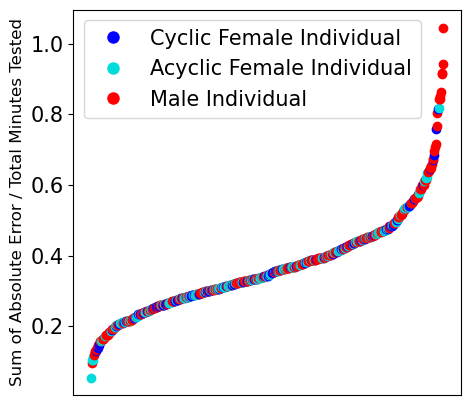


**Supplementary Figure 3.** Average error per minute (sum of the absolute error divided by the total minutes tested - 3995) generated in each individual by linear interpolation in intervals of length 5 to 40 and by the phase-dependent filler in intervals of length 80 to 1440 in simulated missing MET data. If the data were not filled, the sum of absolute error to total minutes tested would result in an average error of at least 0.9.


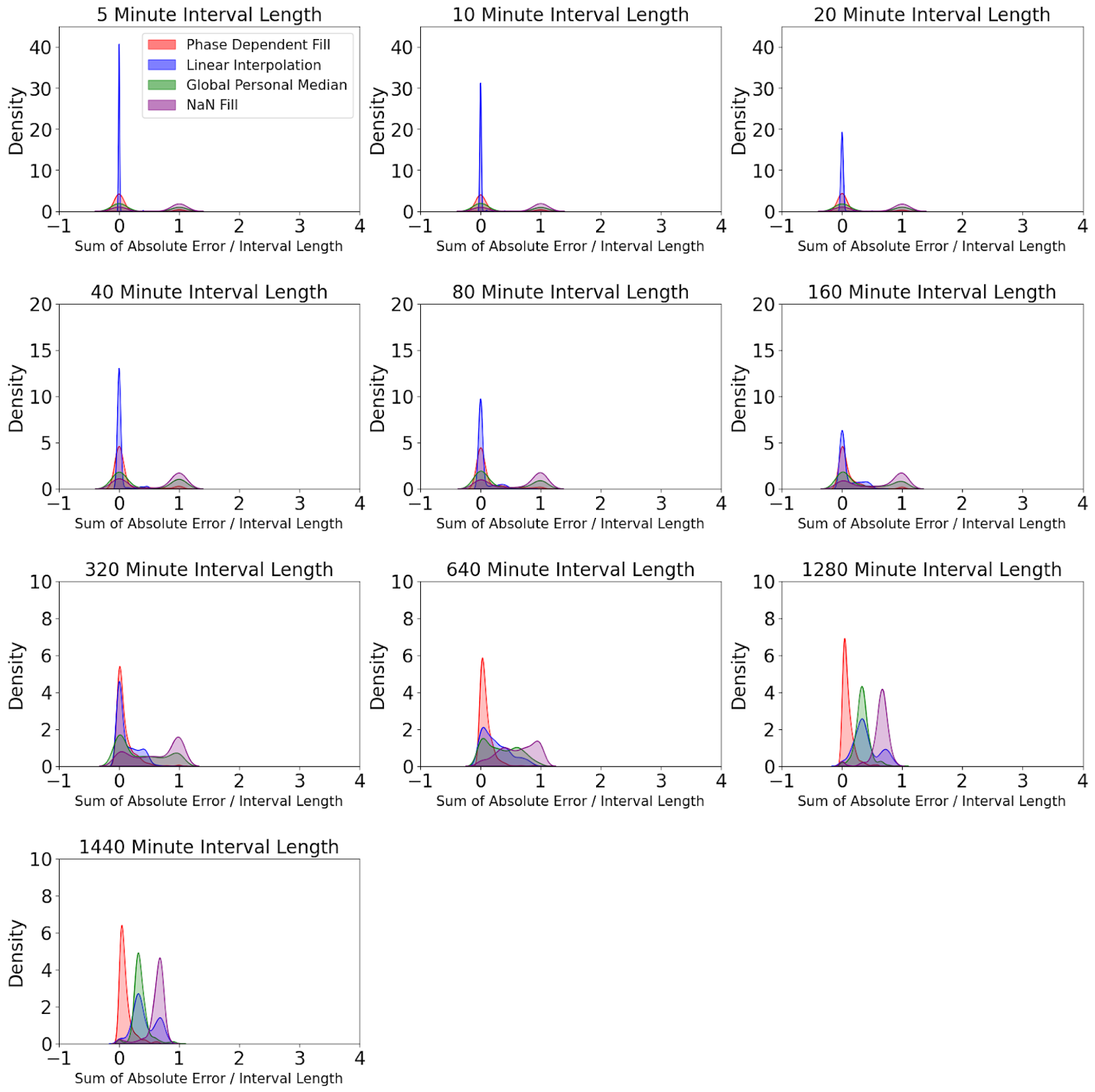
**Supplementary Figure 4.** Kernel density estimate plots of the error generated by each filling method on simulated missing sleep state data. Error is shown as the sum of the absolute error divided by the interval length, or average error per minute. Each distribution is composed of exactly 596 measurements, one of each interval length for every individual.


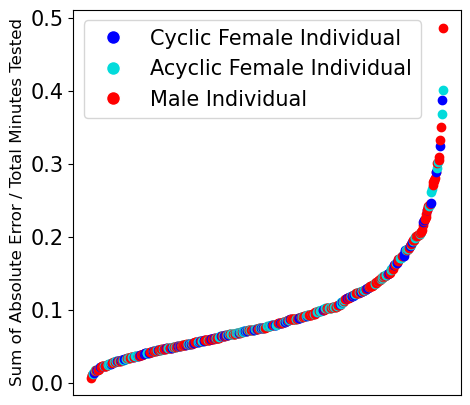


**Supplementary Figure 5.** Average error per minute (sum of the absolute error divided by the total minutes tested - 3995) generated in each individual by linear interpolation in intervals of length 5 to 320 and by the phase-dependent filler in intervals of length 640 to 1440 in simulated missing sleep state data. If the data were not filled, the sum of absolute error to total minutes tested would result in an average error between 0 and 1.
